# A method using artificial neural networks to morphologically assess mouse blastocyst quality

**DOI:** 10.1186/2055-0391-56-15

**Published:** 2014-08-30

**Authors:** Felipe Delestro Matos, José Celso Rocha, Marcelo Fábio Gouveia Nogueira

**Affiliations:** Laboratory of Applied Mathematics (Laboratório de Matemática Aplicada - MaAp), School of Sciences and Letters (Faculdade de Ciências e Letras – FCL) São Paulo State University (Universidade Estadual Paulista – Unesp), Assis, Brazil; Laboratory of Embryo Micromanipulation (Laboratório de Micromanipulação Embrionária - LaMEm), FCL/Unesp, Assis, Brazil

**Keywords:** Embryology, Quality, Assessment, Artificial neural networks, Mice, Software, Blastocyst

## Abstract

**Background:**

Morphologically classifying embryos is important for numerous laboratory techniques, which range from basic methods to methods for assisted reproduction. However, the standard method currently used for classification is subjective and depends on an embryologist’s prior training. Thus, our work was aimed at developing software to classify morphological quality for blastocysts based on digital images.

**Methods:**

The developed methodology is suitable for the assistance of the embryologist on the task of analyzing blastocysts.

The software uses artificial neural network techniques as a machine learning technique. These networks analyze both visual variables extracted from an image and biological features for an embryo.

**Results:**

After the training process the final accuracy of the system using this method was 95%. To aid the end-users in operating this system, we developed a graphical user interface that can be used to produce a quality assessment based on a previously trained artificial neural network.

**Conclusions:**

This process has a high potential for applicability because it can be adapted to additional species with greater economic appeal (human beings and cattle). Based on an objective assessment (without personal bias from the embryologist) and with high reproducibility between samples or different clinics and laboratories, this method will facilitate such classification in the future as an alternative practice for assessing embryo morphologies.

## Background

Since the first techniques for multiple ovulation embryo transfer (MOET) and in vitro fertilization (IVF) were successfully developed in mammals, a clear, direct relationship between embryo quality and gestation rate following embryo transfer to recipient females has been established. Embryos that are morphologically classified as high-quality yield higher gestation rates [1, 2]. Thus, the field requires a system that can standardize the elements used to categorize embryos into different quality grades, which is an indirect indication of viability.

Currently, a four-grade system is used for cattle: excellent, good, fair and poor [3, 4]. This system is based on visual analyses (subjective and qualitative) of embryo morphology, which are commonly performed through optical microscopy (stereomicroscopy). The technique depends on an embryologist’s experience and accuracy in analyzing and categorizing samples from the most obvious variables to the nuances that indicate an embryo is more or less apt to develop. For this classic embryo morphology analysis, the variables are not measured objectively; thus, the method is subjective and has limited reproducibility [5]. As a result, the same embryo measured by different experts may be classified with different quality grades. Such inconsistency is typical for adjacent grades, such as good and excellent embryos [6].

Various alternative methods have been developed to solve the subjectivity problem in embryo morphological analyses [7–10]. The most significant such methods include in vitro embryo culture [7], blastomere membrane integrity analysis [7], embryo metabolism analysis [7], cellular respiration measurements [8], electron microscopy analysis [9] and zona pellucida birefringence indices [10]. However, no method has provided a definitive solution for measuring embryo quality, and it is necessary to develop such fast, non-invasive and objective methods [3, 7]. In addition, such methods can be prohibitively expensive for widespread use. Thus, despite its subjectivity and limited reproducibility, visual morphology analysis persists for embryo quality determinations.

Herein, we validate a method for morphological analysis that is more precise, wherein information is extracted from two-dimensional digital embryo images and the images are subsequently analyzed using software. The software (Blasto4Q) is based on artificial neural networks (ANNs), which is an artificial intelligence technique that solves non-linear problems with interconnected variables [11–13]. ANNs have been applied to various areas, including administrative aids [14] and stock market index predictions [15]. An ANN is a system that solves problems by simulating biological neurons. The neurons in an ANN (also, “perceptrons”) must receive training data to learn and generalize output based on an input dataset. Once it is properly trained, an ANN can generate predictions without a pre-established classification [11, 12, 14, 16]. Therefore, an ANN is an intelligent system that can solve a complex problem based on assisted learning.

## Methods

The embryos used herein were products from other ongoing projects in the Laboratory of Embryo Micromanipulation (Laboratório de Micromanipulação Embrionária - LaMEm, UNESP, Assis). The experiments in this laboratory were developed for applied embryology and embryo micromanipulation. Mouse (Mus musculus) embryos from the Swiss-Webster and C57BL/6 EGFP strains were used. The embryos originated from projects that were approved by the Ethical Commission on Animal Use (Comissão de Ética no Uso de Animais – CEUA) in the School of Sciences and Letters of Assis (protocols 007/2010, 015/2011 and 026/2011).

The embryo images were collected using an Eclipse Ti inverted microscope (Nikon, Japan) coupled to a Digital Sight (Nikon, Japan) camera, which was controlled by the NIS - Elements Advanced Research 3.0 (Nikon, Japan) software. The image may contain one or several embryos, as long as the individual embryo shape is not affected. The magnification of the image capture, as well the resolution of the image file can be chosen by the user, as the software uses on ratios between values.

For the purpose of organizing the database, each embryo was labeled using a code that included one number and letter; the number identified the image in the database, and the letter identified the embryo in the image. Such labeling was performed using the GIMP 2.6.11 software.

**Figure 1:**
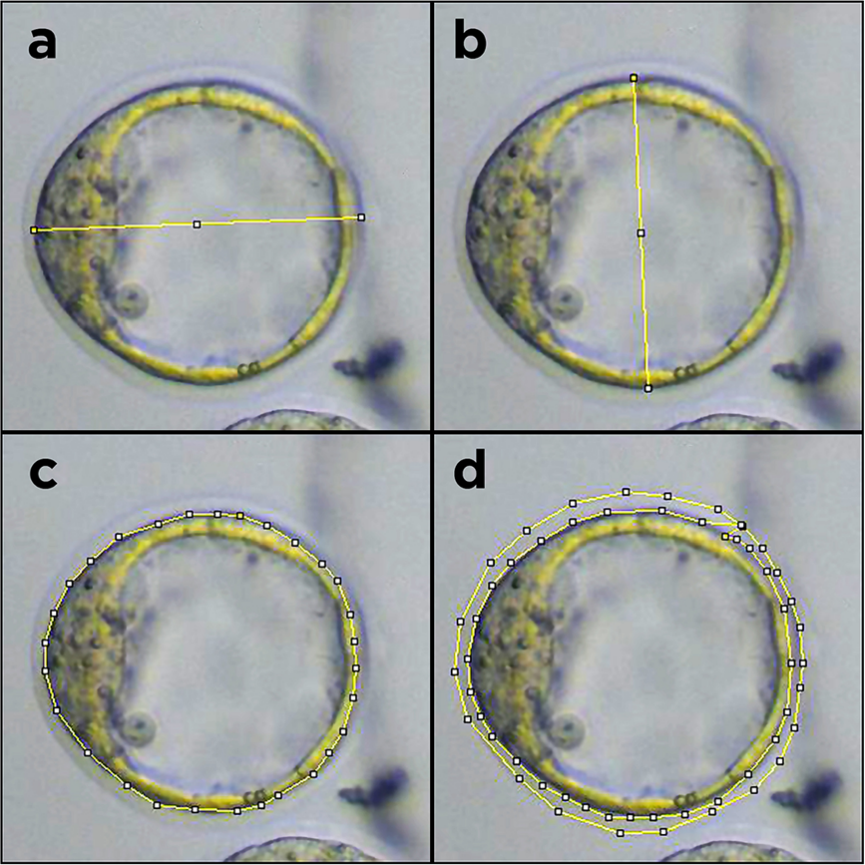
**Example measurements from ImageJ, which represent the points required to determine the mean embryo diameter (images a and b), embryo area (image c) and zona pellucida area (image d).**

All the measurements on the image were made using the software ImageJ 1.45 s. First the previously captured image is loaded on the interface. The user then must use the “Straight Lines” and “Polygon” tools to assess the proportions from the embryo, as indicated on Figure 1.

The data were collected from the embryos using the ImageJ 1.45 s software. The following data were the basis for calculating the following variables: smallest embryo diameter (ED1), largest embryo diameter (ED2), smallest zona pellucida diameter (ZPD1), largest zona pellucida diameter (ZPD2), embryo area (EA), zona pellucida area (ZPA), dead cell area (DCA), live cell area (LCA), embryo color density (ECD), total color density (CDtotal) and zona pellucida color density (ZPCD).

When the perivitelline space was absent (expanded blastocyst), the first two vectors, which determine the embryo and zona pellucida areas, were sufficient to discern the zona pellucida color density using the following ratio (notably, the area vector also yields the mean color density for the area).


Mz is the mean color for the zona pellucida, Mez is the mean color for the zona pellucida and embryo, Aez is the area that comprises the zona pellucida and embryo, Me is the embryo mean color, and Ae is the embryo area.

Compared with manually selecting the zona pellucida area, this formula yielded a more rapid and efficient process for determining the zona pellucida color density (ZPCD); this formula was incorporated into Blasto4Q.

The ANN creation process, the algorithm that determines the best architecture and the graphical user interface were developed using MATLAB R2011a and the Artificial Neural Network Toolbox [17]. To better understand such processes and their adaptation to the overarching problem and its particularities, the standard models for constructing an ANN (ready and available in the “toolbox”) were not used, and the ANN was constructed using the MATLAB metalanguage.

## Results

### Embryo collection and image capture

Training the ANN herein required a database with embryo images that can be properly classified and analyzed. The animals used were superovulated Swiss-Webster mice, and the structures were harvested (eggs and viable or degenerate embryos) 3.5 days after copulation (consistent with the blastocyst stage; see the Methods section for more details). After the embryos were collected, those with viable cells were grouped and photographed using a digital image capture system. We only used embryos that were viable during the blastocyst stage (including the early blastocyst, blastocyst and expanded blastocyst stages) and images in which the blastocyst was largely in focus. Thus, the ANN was trained using 98 images.

### Embryo classification

The conventional morphological classification system 3 was used to classify the selected images as excellent, good, fair or poor grade. Of the 98 embryos, 40 (40.8%) were classified as excellent, 46 (46.9%) were good, 8 (8.2%) were fair, and 4 (4.1%) were poor. These data were used to train the ANN, which generated 4 distinct outputs, one for each embryo quality grade.

### Definitions for the ANN variables

We determined the features that were desirable for assessing embryo quality because such features should be discerned using only static two-dimensional images. Thus, we used the biological aspects of embryo morphology, experience from the quality-assessing embryologist and computational techniques for image processing; the following 12 variables were isolated.

#### Stage of embryonic development (SED)

The embryo’s development stage is critical for the ANN to correctly manage the additional variables given the morphological differences in embryos throughout development before they are implanted (from the zygote to blastocyst stages). For the ANN used herein, this variable indicates whether an embryo is at an early blastocyst, blastocyst or expanded blastocyst stage.

#### Days after copulation (DAC)

The DAC variable is used to compare the SED and the ideal stage for the time elapsed since fertilization. Depending on the DAC value, the embryo should be at a specific stage. Thus, this variable is used to characterize consistency between the level of embryo development assessed with the ideal development level.

#### Ratio between developmental stage and group mean (RGM)

The developmental stage of an embryo relative to other embryos in the same harvest must be considered. For example, an embryo’s stage may be delayed compared with its DAC value, which the ANN may penalize. However, if the other embryos are similarly delayed, such penalization may be reduced or eliminated.

Thus, the RGM was determined using the following formula.


SEDgroup is the mean embryonic stage for the remaining embryos in the same harvest.

Therefore, values greater than 1 indicate that the embryo is at a more advanced stage relative to its group, while values less than 1 indicate that an embryo is at a delayed stage relative to its group.

#### Ratio (RMD) for the mean embryo diameter (ED) and mean zona pellucida diameter (ZPD)

Information on embryo morphology is key to generating data using the ANN. The data generated are dimensionless to avoid scaling problems. Thus, distances can be measured using pixels, micrometers or millimeters, and a ratio for the embryo and zona pellucida measurements is used. This metric was selected because the zona pellucida diameter and embryo diameter ratio is highly consistent. The following formula defines this ratio:


ED is the embryo diameter, and ZPD is the zona pellucida diameter.

Both the ED and ZPD were determined using the means for the largest and smallest embryo and zona pellucida diameters, respectively.

#### Ratio for the live cell area and total area (RLC)

This ratio is calculated to determine the proportion of live cell area in an embryo’s (LCA) total area, which is defined by the outer border for the zona pellucida (ZPA).


#### Ratio (RDC) between the dead cell area (DCA) and live cell area (LCA)

This ratio was created so that the ANN considers dead cells in the embryo for quality analysis.


Greater RDC values indicate a larger proportion of dead cells in the embryo, which negatively impacts its quality.

#### Ratio (RCD) between the embryo color density (ECD) and zona pellucida color density (ZPCD)

Embryo color is another important factor for analysis because it is directly affected by cell density and viability. This variably is highly dependent on the conditions used to photograph the image, including both illumination and the camera control software settings. However, using the ratio between the embryo color (ECD) and zona pellucida color (ZPCD) compensates for such variations.


RCD values less than 1 indicate that an embryo is lighter than its zona pellucida, while values greater than 1 indicate a darker embryo.

The color intensity (ECD or ZPCD) is measured as the mean brightness value for each pixel in a particular area. This value ranges from 0 (completely black) to 255 (completely white).

#### Ratio (RER2) between the embryo roundness (ER) and zona pellucida roundness (ZPR) squared

An additional factor that may be a good indicator for quality is comparing an embryo’s roundness with the typical level of roundness at a blastocyst embryo stage. Mathematically, roundness is determined using the following formula:


An ideal circle has the value 1. As the value approaches 0, its shape is less similar to a circle.

Thus, roundness is defined as follows:


ER is the embryo roundness, and ZPR is the zona pellucida roundness.

Because the zona pellucida is stable and round, a value near 1 indicates a round embryo, while values near 0 indicate low roundness.

However, in practice, the values are always near 1; thus, it is difficult for the ANN to assign different features to round or less round embryos. To solve this problem, RER2 (ratio of roundness squared) was used to numerically emphasize small differences in roundness. Notably, rounder embryos trend towards 1 because it is the upper limit.

#### Sharp edges macro (EDG)

The input variables must numerically indicate the visual morphological features for an embryo. However, the aforementioned variables cannot represent an embryo’s roughness or granularity. Thus, a macro (series of automatic operations) was developed for the ImageJ software (further details in the Methods section) to identify and count the contrast regions in the embryo to numerically represent this visual feature. The macro is referred to as “Sharp Edges” because it uses the basic operations sharpen and find edges.

#### Ratios for blastocoel area, color density and roundness (RBA, RBCD and RBR)

Because only blastocysts were used, blastocoel features were also included as input variables. Blastocoel area, color density and roundness were used, and a ratio was established with the respective variable for the embryo. Blastocoel roundness was squared for the aforementioned rationale regarding embryo roundness.


### Data extraction and standardization

The data required to calculate the above-described variables must be extracted from the embryo images; thus, the ImageJ software was used. ImageJ is a free multifunctional image processing software that facilitates measurements using selected points in an image [18]. Figure 1 demonstrates how such selections were generated.

However, the output data from ImageJ cannot be directly used. Such data are the basis for calculating the variables that will be used to train the ANN and for quality analyses using Blasto4Q.

For example, the four measurements used to calculate the variable RMD included the largest and smallest diameters for both the embryo and zona pellucida. Similar procedures were used to collect the input variables (see the Methods section for further details).

### Developing the ANN architecture

The structure of an ANN includes various elements, such as the numbers of neuron layers and neurons in each layer as well as their transfer functions and the network training function. Although it is important to correctly establish such factors to optimally develop an ANN, there is not a standard protocol to determine the best architecture [19].

**Figure 2:**
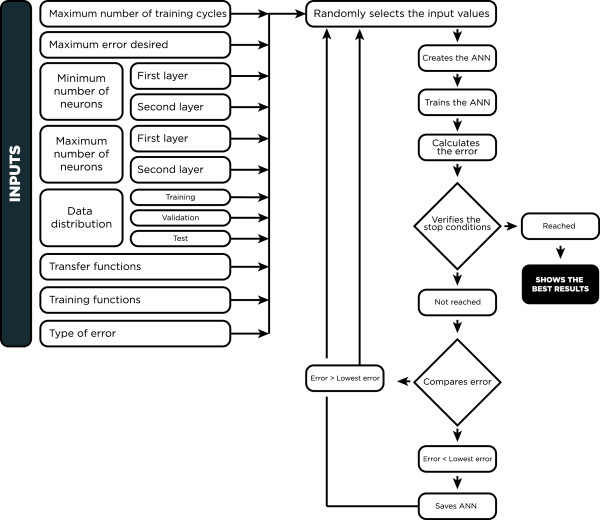
**Flowchart for the algorithm used to construct the ANN architecture.** Initially, the variables used to create the various ANN configurations were included. The program randomly selects values for each variable and generates an ANN, which is trained using the database; the data are divided into training, validation and test sets in accordance with the initial program selection. This process is repeated; the program compares the error for a network with previous networks, and the ANN with the lowest error is saved. When it encounters a stop variable, the program ends the cycle and displays the best result.

To address this problem, an algorithm was developed that automatically tests various combinations and structures to determine the best result. The flowchart for this algorithm is shown in Figure 2.

This algorithm was executed using Matlab software with the stop condition 10,000 cycles; the range 5 to 20 neurons for the first and second layers; and randomly selected tansig, logsig and purelin transfer functions as well as trainlm, trainscg and traingdx training functions. The error was calculated using a confusion matrix (incorrect classification percentage). The ANN was developed for the backpropagation algorithm. Table 1 shows the best results generated.

**Table 1:** **Results from the algorithm used to determine the best ANN architecture**

Error from training	12.07%
Error from validation	30.00%
Error from test	25.00%
Error from all data	18.37%
Mean Square Error (MSE)	0.023206
Training Data	60%
Validation Data	20%
Test Data	20%
Number of neurons in the first layer	18
Number of neurons in the second layer	13
Function in the first layer	purelin
Function in the second layer	logsig
Training function	trainscg

**Figure 3:**
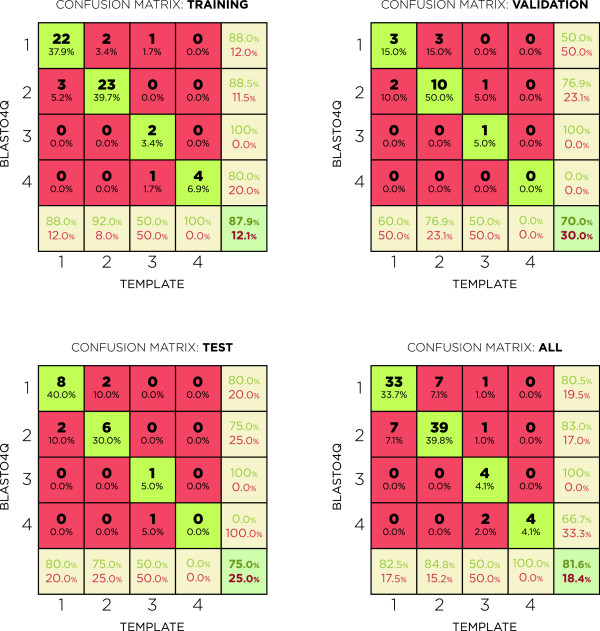
**Confusion matrix.** Each matrix includes the hits for each ANN processing group (training, validation and test). The embryos were distributed vertically in accordance with the template and horizontally in accordance with the ANN classification. Thus, the diagonal for each matrix (highlighted in green) shows the ANN hits, which is the number of cases where the ANN is consistent with the template. The final item (bottom right in each matrix) shows the percentage of hits (highlighted in green) and error (highlighted in red) for the ANN.

Thus, herein, the best ANN architecture was a network that included 18 neurons in the first layer with a purelin transfer function (linear function) and 13 neurons in the second layer with a logsig transfer function (logistic function). The algorithm selected trainscg (Scaled Conjugate Gradient Algorithm) for the training function [20]. The data used to train, validate and test the ANN are summarized in Figure 3. Each confusion matrix shows the relationship between the real data (template) and the data simulated by the network.

### Validation

The data processed by the ANN were divided into three classes: training (60% of the data), validation (20%) and test (20%). Each dataset was randomly generated each time the ANN was trained. The training data were effectively used to teach the ANN. The validation data were used to avoid overfitting by the ANN; data may be overfit when a network is excessively trained with a dataset and incorporates input noise [11]. The final test dataset was not provided to the ANN during the training phase; thus, the test data were used to verify whether the ANN was effectively trained because the network is used to classify a “novel” dataset (without prior access to the data) after training and validation.

Table 2 shows the ANN results for the test data. In this table, the ID column identifies the embryo in the database. The error column was calculated by subtracting the ANN-assigned quality from the template-generated quality (embryologist assessment), where 0 is a hit, +1 indicates that the ANN assigned a lower quality score than the template, and −1 indicates that the ANN assigned a higher quality score than the template.

**Table 2:** **Results from the ANN for the test data**

ID	ANN outputs				Quality ANN	Quality template	Error
	1	2	3	4			
003D	0.11	0.84	0.03	0.01	2	1	1
004E	0.45	0.64	0.00	0.00	2	2	0
004 F	0.83	0.10	0.01	0.01	1	2	−1
004G	0.86	0.09	0.01	0.01	1	2	−1
008C	0.53	0.40	0.11	0.00	1	1	0
008D	0.21	0.62	0.05	0.00	2	2	0
011A	0.75	0.27	0.02	0.00	1	1	0
012C	0.47	0.48	0.11	0.00	2	2	0
013G	0.84	0.13	0.01	0.00	1	1	0
015A	0.55	0.47	0.05	0.00	1	1	0
016B	0.94	0.04	0.02	0.00	1	1	0
016D	0.37	0.63	0.02	0.01	2	2	0
016E	0.74	0.07	0.21	0.00	1	1	0
017 F	0.21	0.85	0.02	0.00	2	1	1
024C	0.22	0.79	0.03	0.00	2	2	0
027B	0.63	0.04	0.34	0.00	1	1	0
027 J	0.00	0.20	0.18	0.90	4	3	1
028I	1.00	0.00	0.00	0.01	1	1	0
029 F	0.00	0.58	0.58	0.15	2	2	0
029E	0.00	0.27	0.51	0.14	3	3	0

The ANN provided a correct prediction (its analysis was equal to the template) in 75% of the cases (15 hits from 20 test samples). Of the 5 incorrectly classified cases, the ANN assigned a quality score 1 grade above the template (for example, a grade 2 embryo was classified as grade 1) in 3 cases and at a lower grade in 2 cases (for example, a grade 1 embryo was classified as grade 2).

The 75% hit rate indicates the cases where the ANN generated the same value as the template used to train the ANN. However, embryo classification can vary depending on the evaluator, who can categorize the same embryo at two or three different but adjacent grades [6]. In reality, this is common, primarily where the image does not have a good focal plane for analyses or the embryo assessed is at the border between two quality grades, such as excellent and good.

Therefore, another analysis was performed using the test data and the same embryologist that performed the initial classification, who reassessed the embryos blind to both their prior classification and the ANN’s classification. In the same analysis, the evaluator indicated the possible quality grades that may correctly classify the embryo (see Table 3).

**Table 3:** **Comparison for the original assessment, reassessment and ANN results**

ID	Original assessment	Reassessment	Possible grades				ANN Result	Reassessment error	ANN error
003D	1	1	1	2			2	0	1
004E	2	2	1	2	3		2	0	0
004 F	2	1	1	2			1	−1	−1
004G	2	2	1	2			1	0	−1
008C	1	1	1	2			1	0	0
008D	2	2		2	3		2	0	0
011A	1	1	1	2			1	0	0
012C	2	2		2	3		2	0	0
013G	1	1	1	2			1	0	0
015A	1	2	1	2			1	1	0
016B	1	1	1	2			1	0	0
016D	2	2	1	2			2	0	0
016E	1	2	1	2			1	1	0
017 F	1	2	1	2			2	1	1
024C	2	2	1	2			2	0	0
027B	1	2		2	3		1	1	0
027 J	3	3			3	4	4	0	1
028I	1	2	1	2			1	1	0
029 F	2	3		2	3		2	1	0
029E	3	3			3		3	0	0

These results were compared with the original template and ANN classifications, which are shown in Table 3. Comparing the reassessment by the embryologist, the ANN assessment and the original assessment showed that, while the ANN incorrectly classified five cases (25%), seven cases (35%) were incorrectly classified upon reassessment. This finding demonstrates that the human factor (assessment by an embryologist) was responsible for limited reproducibility of the assessments. Moreover, the three classifications were within the possible correct grades in almost each case (except for the image 027B, for which the reassessment was inconsistent with the original template and ANN classification; it was correct in the original assessment but was incorrectly classified upon reassessment). The image 029E presented an interesting case, for which fair was the only possible grade, and each assessment was consistent.

The 75% hit rate indicates the cases where the ANN is consistent with the template. However, the blind reassessment test presents the possible correct grades for the test embryos. A new analysis was performed using these possible grades for the error analysis (Table 4), not the original template, from which we generated a 95% hit rate with only 1 incorrect classification.

**Table 4:** **All possible quality grades considered in a single embryo assessment**

ID	Possible scores				Blasto4Q	Classification
003D	1	2			2	Correct
004E	1	2	3		2	Correct
004F	1	2			1	Correct
004G	1	2			1	Correct
008C	1	2			1	Correct
008D		2	3		2	Correct
011A	1	2			1	Correct
012C		2	3		2	Correct
013G	1	2			1	Correct
015A	1	2			1	Correct
016B	1	2			1	Correct
016D	1	2			2	Correct
016E	1	2			1	Correct
017F	1	2			2	Correct
024C	1	2			2	Correct
027B		2	3		1	Incorrect
027J			3	4	4	Correct
028I	1	2			1	Correct
029F		2	3		2	Correct
029E			3		3	Correct

#### Blasto4Q

The software Blasto4Q was developed as the final result herein. This software is fully functional and can be installed on any computer running the Windows operating system (the compatibility test was conducted using Windows 7; other versions of Windows have not been tested). Matlab Compiler Runtime (MCR) and Java Virtual Machine (JVM) must be installed by the end-user; they can be acquired from their respective companies.

**Figure 4:**
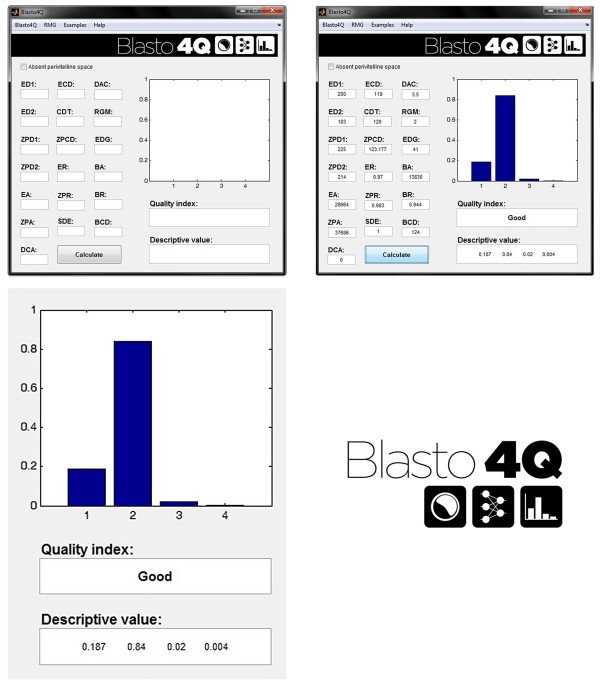
**Blasto4Q graphical user interface.** From left to right, top to bottom: the initial screen of the program; input data and the result; details for the output; and software logo.

Creation and development of this system would be meaningless without a practical, fast and efficient way for the end-user to apply the program. Thus, a graphical user interface (GUI) was developed, as shown in Figure 4.

Blasto4Q provides results in the following three ways (see Figure 4).

- A bar chart that represents each ANN output (the 4 quality grades); the height of each bar is determined by the output value magnitude.
- A quality index using the network’s highest output value; the possible results are excellent, good, fair and poor.
- A descriptive vector that is output vector for the ANN; this vector represents the values for the four neurons in the output layer (“Excellent”, “Good”, “Fair” and “Poor”).

## Discussion

Based on the work of this study, we established an alternative method to classify blastocyst morphology in mice. We used an ANN based on data from static, two-dimensional digital images and combined with a graphical user interface to generate a proposed method for a quantitative, objective and highly reproducible assessment. Embryo morphological classification is important for numerous laboratory techniques, which range from basic methods to assisted reproduction applications. Success rates (gestation) for associated biotechniques (cryopreservation, biopsy, embryo splitting and microinjection, among others) can be inferred using this technique, and the embryos used for scientific experiments can be standardized. However, the method used to classify mammalian embryos (e.g., from human beings, horse, cattle, rats and mice) has always been based on a subjective assessment by an evaluator. Despite the standards for quality grades and the morphological characteristics that characterize each grade, inconsistencies are common for classification by different evaluators, even if they are experienced embryologists [6].

Alternative models for morphological classification with greater objectivity have been developed [7–10]. However, such methods should be fast, low-cost, high-resolution and non-invasive [3, 7]. In particular, it is necessary to exercise extreme caution to minimize iatrogenic damage to the embryo by the technique (e.g., through prolonged exposure to non-ideal conditions or excessive handling). Thus, conventional morphological assessments are widely used despite the limited subjectivity (due to the problem with reproducibility and accuracy). Blasto4Q is a reliable morphological analysis technique because the result will always be the same for a given input after the ANN is trained (objectivity and reproducibility).

The Blasto4Q software facilitates rapid assessment with minimal interference in embryonic development because only a single digital image of the embryo is necessary, which requires a microscopy system (inverted or not) coupled to a digital image capture system. The embryos are stored under favorable conditions inside CO_2_ incubators when the analyses are performed (data collection and simulation through Blasto4Q). Additionally, the analyses performed by the software are more detailed and produce more data than from an embryologist. Although embryologists may have experience distinguishing among subgrades, one standard grade must be assigned (e.g., an embryo that is grade “1.5” would be either grade 1 or grade 2, depending on the evaluator’s analysis). Because a result from Blasto4Q is the descriptive vector, each embryo can be given an “identity” or values that represent the probability that an embryo will be classified in each of the four possible grades.

The blastocyst stage was used for preliminary tests because it is important for commercial in vitro bovine embryo production and due to its growing relevance in assisted human reproduction compared with pre-compaction embryonic stages. Different embryologists can provide a template for ANN training in accordance with the specific classifications used for embryo morphological assessment. Thus, this process has a high potential for applicability because it can be adapted to additional species with greater economic appeal (human beings and cattle). Based on an objective assessment (without personal bias from the embryologist) and with high reproducibility between samples or different clinics and laboratories, this method will facilitate such classification in the future as an alternative practice for assessing embryo morphologies.

## Conclusions

This process has a high potential for applicability because it can be adapted to additional species with greater economic appeal (human beings and cattle). Based on an objective assessment (without personal bias from the embryologist) and with high reproducibility between samples or different clinics and laboratories, this method will facilitate such classification in the future as an alternative practice for assessing embryo morphologies.
